# Membrane Proteins in Trypanosomatids Involved in Ca^2+^ Homeostasis and Signaling

**DOI:** 10.3390/genes9060304

**Published:** 2018-06-19

**Authors:** Srinivasan Ramakrishnan, Roberto Docampo

**Affiliations:** 1 Center for Tropical and Emerging Global Diseases, University of Georgia, Athens, GA 30602, USA; sri@uga.edu; 2 Department of Cellular Biology and Center for Tropical and Emerging Global Diseases, University of Georgia, Athens, GA 30602, USA

**Keywords:** calcium, *Trypanosoma*, *Leishmania*, mitochondria, acidocalcisome

## Abstract

Calcium ion (Ca^2+^) serves as a second messenger for a variety of cell functions in trypanosomes. Several proteins in the plasma membrane, acidocalcisomes, endoplasmic reticulum, and mitochondria are involved in its homeostasis and in cell signaling roles. The plasma membrane has a Ca^2+^ channel for its uptake and a plasma membrane-type Ca^2+^-ATPase (PMCA) for its efflux. A similar PMCA is also located in acidocalcisomes, acidic organelles that are the primary Ca^2+^ store and that possess an inositol 1,4,5-trisphosphate receptor (IP_3_R) for Ca^2+^ efflux. Their mitochondria possess a mitochondrial calcium uniporter complex (MCUC) for Ca^2+^ uptake and a Ca^2+^/H^+^ exchanger for Ca^2+^ release. The endoplasmic reticulum has a sarcoplasmic-endoplasmic reticulum-type Ca^2+^-ATPase (SERCA) for Ca^2+^ uptake but no Ca^2+^ release mechanism has been identified. Additionally, the trypanosomatid genomes contain other membrane proteins that could potentially bind calcium and await further characterization.

## 1. Introduction

Trypanosomatids are a group of unicellular parasitic organisms. This group includes two important genera: *Trypanosoma* and *Leishmania*, with several species that cause severe diseases in humans. *Trypanosoma brucei* and *Trypanosoma cruzi* cause African sleeping sickness and Chagas disease, respectively. *Leishmania* species are the causative agents for cutaneous, mucocutaneous and visceral leishmaniases. *T. brucei* is an extracellular parasite that replicates in the digestive system of *tsetse* flies as procyclic form (PCF) and in the tissue fluids and blood of mammals as bloodstream form (BSF). Similar to *T. brucei*, *T. cruzi* and *Leishmania* spp. replicate extracellularly in the midgut of their insect vectors as epimastigotes and promastigotes, respectively. However, once in the mammalian host, they both replicate intracellularly as amastigotes. While *Leishmania* amastigotes propagate further by infecting other macrophages, *T. cruzi* amastigotes convert to trypomastigotes before host cell lysis and enters the circulatory system to infect other nucleated cells.

Ca^2+^ signaling pathways in trypanosomatids are highly divergent from those in the mammalian hosts they infect. As a result, these pathways have been researched thoroughly for identification of potential targets for drugs, vaccines, and diagnostic tools. In the process, we have learned that these parasites possess many unique aspects that are central to their Ca^2+^ signaling network.

Both *Trypanosoma* and *Leishmania* species possess a single mitochondrion. Similar to mammalian cells, the mitochondrial Ca^2+^ uptake is mediated by a mitochondrial Ca^2+^ uniporter (MCU) complex. The discovery that trypanosomes have a MCU with similar physiological properties as the mammalian uniporter [1,2] was essential [3] for the discovery of the genes encoding the gatekeeper mitochondrial calcium uptake 1 (MICU1) [4] and the pore (MCU) [5,6] of the channel. MCU is indispensable for parasite growth and infectivity [7] while the channel is not essential in some mouse strains [8].

Moreover, while mammalian cells use the endoplasmic reticulum (ER) as a main Ca^2+^ storage site, trypanosomatids store most of their Ca^2+^ in an acidic store named the acidocalcisome [9]. Interestingly, while the Ca^2+^ export channel, inositol 1,4,5-trisphosphate receptor (IP_3_R), localizes to the ER in mammals, it localizes to acidocalcisomes in trypanosomatids [10,11]. In this review, we highlight differences in Ca^2+^ homeostasis and signaling pathways in *Trypanosoma* and *Leishmania* species as compared to those in their mammalian hosts. We indicate the specific proteins that mediate Ca^2+^ import and export in various organelles and identify proteins that have the potential to bind Ca^2+^ and participate in their overall Ca^2+^ homeostasis.

## 2. Significance of Ca^2+^ Signaling in Trypanosomatids

During evolution, two alkali metals, calcium, and magnesium, were present in high concentrations in the primordial soup. Both these elements had similar chemical properties and were chosen to perform many important functions in evolving cells and organisms. However, for inter and intracellular communication, calcium was chosen over magnesium as an important signaling regulator. Both calcium and magnesium can form stable cations by readily losing two electrons. However, calcium, with a larger ionic radius, loses its outermost electrons more easily than magnesium. This enabled calcium to readily participate in multiple chemical reactions that led to the evolution of life. The larger ionic radius also provided calcium with larger polarizability. This meant that calcium ions were highly flexible and therefore able to interact with sites of irregular geometry in various proteins [12]. Additionally, phosphate-based energetics, which also evolved early during evolution also preferred calcium as the main signaling ion due to its lower charge density [13]. Therefore, during evolution calcium emerged as an important cellular messenger. In eukaryotes, many important processes such as cell division, metabolism, motility, regulation of cell death, etc., are all governed through Ca^2+^ signaling. Similar to other eukaryotes, Ca^2+^ plays a ubiquitous role in trypanosomatids as well.

A variety of proteins that could be modulated by Ca^2+^ have been found in trypanosomatid genomes. Putative Ca^2+^-calmodulin dependent kinases have been identified [14]. In eukaryotes, calmodulin (CaM) kinases participate in gene transcription, translation, ion channel regulation as well as cell death processes [15]. In *T. cruzi* a CaM kinase II, which is activated by reactive oxygen species [16], was shown to play a role in heme-induced proliferation of parasites [17]. A CaM kinase from *T. cruzi* was also purified and its enzymatic characteristics were analyzed in detail [18,19].

Ca^2+^ activates the *T. cruzi* phosphoinositide phospholipase C (PI-PLC). This enzyme localizes to the plasma membrane and is upregulated during trypomastigote to amastigote conversion, indicating a potential role for Ca^2+^ in parasite differentiation [20]. This is supported by the observation that cytosolic Ca^2+^ concentration changes occur during differentiation of *T. cruzi* epimastigotes to metacyclic trypomastigotes [21]. Similarly, cytosolic Ca^2+^ concentration changes also occur during differentiation of *T. brucei* bloodstream forms to procyclic forms [22].

A *T. cruzi* adenylyl cyclase, which interacts with a paraflagellar rod protein is known to be activated by Ca^2+^ indicating a possible role for Ca^2+^ signaling in parasite motility [23]. The role for Ca^2+^ in parasite motility is further corroborated by the parasite centrins. Centrins are well known Ca^2+^ binding proteins. Out of the five centrins found in *T. brucei*, three localize to the flagellar basal body [24]. They play a role in flagellar motility [24] but are also necessary for organelle segregation [25] and cell division [26]. Similarly, a centrin in *Leishmania donovani* localizes to the basal body and is indispensable for basal body duplication and cell division [27,28] thereby highlighting how Ca^2+^ binding proteins could be playing a pivotal role in trypanosomatid motility and cell division.

Another protein that is activated by Ca^2+^ in trypanosomatids is the *T. cruzi* calcineurin [26,29]. Inhibition of calcineurin activity or downregulation of its expression inhibits parasite invasion of HeLa cells suggesting a role for Ca^2+^ in parasite invasion [29]. In support of its role in invasion, significant increase in intracellular Ca^2+^ is observed during parasites interaction with their host cells [30,31]. Prevention of this Ca^2+^ increase in the parasites results in decreased invasion for both *T. cruzi* [30] and *Leishmania amazonensis* [31]. Additionally, chelation of Ca^2+^ also impairs invasion of host cells [32]. Ca^2+^ has also been proposed to function in parasite osmoregulation [33], maintenance of cytoskeleton [34], and regulation of programmed cell death [35].

## 3. Calcium Transport Proteins in the Plasma Membrane

Several different types of transporters can mediate Ca^2+^ import through the plasma membrane. These include voltage gated Ca^2+^ channels (VGCCs), ligand gated Ca^2+^ channels (LGCCs), transient receptor potential channels (TRPs) and store-operated Ca^2+^ entry channels (SOCEs).

VGCCs are Ca^2+^ channels that respond to membrane depolarization events. Such channels are classified into high voltage (HVA) L, N, P/Q, R—type or low voltage T-type channels depending on their pharmacological and gating characteristics. Sequences of L, N, P/Q, and T type voltage channels from mammalian cells and sequence of the yeast VGCC named ScCch1p were used to search for VGCCs in parasite genomes. Such a search has identified at least one VGCC ortholog in *T. brucei* and *T. cruzi* whereas *Leishmania* spp. seem to harbor two such orthologs [36]. These orthologs from *T. cruzi* and *Leishmania* spp. have not been characterized yet. The *T. brucei* ortholog for VGCC has been shown to localize to the region where the flagellum attaches to the parasite cell body (known as the flagellar attachment zone) and appears to be necessary for flagellar attachment and proper parasite growth in *T. brucei* BSF parasites [37]. However, its role in Ca^2+^ signaling specifically has not been studied. Evidence has been presented on the presence of an sphingosine-stimulated Ca^2+^ channel in the plasma membrane of *Leishmania mexicana* [38] that is different from the nifedipine-inhibited L-type channel also present in these parasites [39].

LGCCs are ionotropic Ca^2+^ channels that open in response to a ligand and have not been reported to exist in protozoan parasites [40].

TRP channels open in response to either physical or chemical stimuli. They have been categorized into six families as TRPC (canonical), TRPM (melastatin), TRPV (vanniloid), TRPA (ankyrin), TRPML (mucolipin) and TRPP (polycystin) family. Among these, at least TRPC, TRPV, TRPA and TRPP have been shown to localize to plasma membrane and permeate Ca^2+^. Searching the parasite genome with either full-length or *N*-terminally truncated isoforms of human TRP proteins revealed two orthologs for TRPML/TRPP type in *Leishmania* spp. and *T. brucei*. Interestingly, this same search revealed four orthologs of TRPML/TRPP type in *T. cruzi* CL Brener strain, which has a hybrid genome [36]. Other strains have at least three distinct orthologs (Table 1). One of the TRPML ortholog from *T. brucei* has been shown to be involved in iron transport [41] while the orthologs from *T. cruzi* were shown to localize to acidic compartments such as reservosomes and lysosomes [42].

In mammalian cells, the SOCE is orchestrated by the activity of stromal interaction molecule (STIM) and ORAI proteins [43]. Both *Trypanosoma* and *Leishmania* spp. seem to lack orthologs for these proteins indicating that SOCE may be absent in trypanosomatids [36].

Ca^2+^ export through the plasma membrane of the mammalian cells is mediated by the co-operative action of a Na^+^/Ca^2+^ exchanger (NCX) and a plasma membrane Ca^2+^ ATPase (PMCA). Orthologs for the Na^+^/Ca^2+^ exchanger NCX are missing in the Excavata supergroup [44]. However, orthologs for PMCA can be found in both *Trypanosoma* and *Leishmania* spp. (Table 1). Four PMCA’s have been identified in *T. cruzi* genome. One of these has two copies and localizes to the plasma membrane as well as to the acidocalcisomes. The other two candidates remain to be characterized [45].

In *T. brucei*, four orthologs for the PMCA can be found. TbPMC1 localizes to acidocalcisomes while TbPMC2 localizes to the plasma membrane. Loss of both these TbPMC proteins affects parasite growth and renders the parasite more sensitive to high levels of extracellular Ca^2+^ [46]. The PMCAs from both *T. brucei* (TbPMC1, TbPMC2) and *T. cruzi* (TcCa1) were able to complement the loss of a vacuolar PMC1 from yeast cells thereby demonstrating the functional capability of these orthologs. An earlier study with vesicles generated from *Leishmania* spp. plasma membrane has indicated the presence of a vanadate sensitive Ca^2+^-ATPase pump in the plasma membrane of these parasites [47]. In agreement with this finding, four orthologs to PMCA can be found in *Leishmania* spp. genomes [40]. However, characterization of these orthologs at the molecular level is still pending. Mammalian PMCAs harbor an auto-inhibitory *C*-terminal calmodulin-binding domain that regulates the activity of PMCA proteins. Although some trypanosomatids apparently lack this *C*-terminal calmodulin binding domain, it has been shown that calmodulin stimulates the plasma membrane ATPase activity in *T. brucei* [40], *T. cruzi* [48] and *L. mexicana* [49]. In this regard, the presence of a CaM-binding domain was recently demonstrated in the *C*-terminal region of the PMCA of *Trypanosoma equiperdum* [50,51], a subspecies of *T. brucei* [52].

## 4. Calcium Transport Proteins in the Membrane of the Endoplasmic Reticulum

Endoplasmic reticulum (ER) is the major Ca^2+^ store in mammalian cells. Uptake of Ca^2+^ into the ER is mediated by the sarco/endoplasmic reticulum Ca^2+^ATPase (SERCA). Orthologs for the SERCA protein have been identified and characterized in trypanosomatids (Table 1). Upon overexpression of the *T. brucei* SERCA ortholog, an increased ATPase activity was observed in the microsomal fractions. This activity was sensitive to thapsigargin [53], a specific inhibitor of the ER Ca^2+^ ATPase. Thapsigargin was also able to release Ca^2+^ from intracellular stores of *Trypanosoma evansi* [54], a subspecies of *T. brucei* [52]. Unlike the *T. brucei* protein, the *T. cruzi* SERCA ortholog is not affected by thapsigargin but clearly localizes to the parasite ER and exhibits Ca^2+^ dependent ATPase activity [55]. A SERCA ortholog has also been characterized in *Leishmania* spp. and appears to be overexpressed in virulent forms of the parasite [31]. Together these data indicate that Ca^2+^ uptake in the ER is a conserved process in trypanosomatids.

Ca^2+^ within the ER is buffered by calcium binding proteins such as calreticulin. *T. brucei* ortholog for calreticulin is yet to be studied. In *T. cruzi* the calreticulin localizing to the ER has been identified and its role in quality control of glycoprotein folding has been characterized in detail [56]. Interestingly, it was observed that upon infection of the mammalian host, this calreticulin translocates from the ER to the parasite surface thereby offering protection against the host complement system [57]. It has also been suggested that the translocated calreticulin binds the host complement component C1 and thereby facilitates parasite invasion [58]. Due to these properties, the *T. cruzi* calreticulin has also been regarded to possess an inhibitory effect on cancerous cells [59]. In *Leishmania* spp., loss of calreticulin affects the parasite secretory pathway and reduces parasite virulence indicating that ER calcium may have an important role in governing virulence of trypanosomatid parasites [60,61].

Ca^2+^ release from ER of mammalian cells is mediated by IP_3_R or ryanodine receptors (RyR). Trypanosomatids lack orthologs for ryanodine receptors but harbor an ortholog for the IP_3_R (Table 1). In *Leishmania* spp., this ortholog has not been characterized. However, in both *T. brucei* [10] and *T. cruzi* [11], the IP_3_R does not localize to the ER indicating that parasites employ a more divergent mechanism of Ca^2+^ release from the ER. Interestingly, another group of proteins termed presenilins have been identified and function as Ca^2+^ leak channels in the mammalian ER [62]. Trypanosomatids harbor orthologs for presenilins in their genome. However, their role in parasite Ca^2+^ signaling is still unconfirmed.

## 5. Calcium Transport Proteins in the Membranes of the Mitochondrion

Ca^2+^ regulates the activity of three dehydrogenases in the mitochondria of mammalian cells. Activation of these enzymes by Ca^2+^ results in increased oxidative phosphorylation and ATP production by the mitochondria [63,64,65]. Accumulation of Ca^2+^ in the mitochondria also acts as a signaling process for regulation of autophagic, apoptotic and necrotic pathways in the cell. A voltage dependent cation channel, VDAC in mammalian cells, mediates transfer of Ca^2+^ through the outer mitochondrial membrane [66]. Further, transfer through the inner mitochondrial membrane occurs by the activity of a mitochondrial Ca^2+^ uniporter complex (MCU complex). The MCU complex in mammalian cells comprises of the following components—(1) MCU, which is the pore forming subunit [5,6]; (2) MCUb which is a dominant negative regulator of the MCU complex [67]; (3) Mitochondrial calcium uptake 1 and 2 (MICU1 and MICU2), which are the gatekeepers of the channel [4,68] and (4) Essential MCU regulator (EMRE) which is indispensable for MCU complex mediated Ca^2+^ uptake [69]. Another protein termed MCUR1 [70] has also been suggested to be a part of the MCU complex and regulate its activity. However, it was proposed that this protein could modulate the mitochondrial membrane potential and therefore exert an indirect effect on the MCU complex [71]. As a result, their inclusion in the MCU complex is under debate [71,72].

Unlike mammalian cells, trypanosomatids harbor a single mitochondrion that runs the length of the cell body of the parasite and has many peculiar characteristics. Despite, such differences the mitochondrial Ca^2+^ uptake mechanism is well conserved between mammalian cells and trypanosomatids. An ortholog for the VDAC protein can be found in *T. brucei*, *T. cruzi* and *Leishmania major* (Table 1). The *T. brucei* VDAC (TbVDAC) has been studied in detail and is essential for parasite growth and mitochondrial ATP production. It has been suggested that TbVDAC acts as a conserved metabolic transporter in the outer mitochondrial membrane of the parasite. However, its direct role in Ca^2+^ import into the parasite mitochondrion was not tested [73]. Later, two other VDAC-like proteins were identified in *T. brucei* by bioinformatic analyses [74]. However, their role in Ca^2+^ import has not been explored either. Similar to the mammalian mitochondria, an MCU complex situated in the inner mitochondrial membrane is involved in driving Ca^2+^ into the trypanosomatid mitochondrial matrix. Orthologs of MCU, MICU1, MICU2 and MCUb have been identified and characterized in both *T. brucei* [10] and *T. cruzi* [75] (Table 1). MCU knockdown in *T. brucei* did not affect the mitochondrial membrane potential, but reduced the mitochondrial Ca^2+^ uptake, increased the AMP/ATP ratio and induced autophagy. Based on these results it has been conceived that the mitochondrial Ca^2+^ uptake is required for activation of dehydrogenases in the mitochondrion of the parasite [7]. Recent development of CRISPR/Cas9 mediated knockout generation has allowed the characterization of MCU and MCUb in *T. cruzi*. Analyses of these mutants indicate that both MCU and MCUb are necessary for Ca^2+^ uptake into the mitochondrion of *T. cruzi*. However, only MCUb was found to be indispensable for parasite growth, metacyclogenesis and infectivity. Additionally, MCUb in *T. cruzi* does not function as a dominant negative regulator of MCU indicating that this protein differs significantly from its mammalian counterpart [75]. MICU1 and MICU2 have not been characterized in these parasites yet. Similar to their proposed mechanism in mammals, they could be playing a regulatory role in trypanosomes as well. However, the absence of a MICU2 ortholog in *Leishmania* spp. indicates that a different and unique role for MICU proteins could exist in trypanosomatid parasites. Orthologs for EMRE and MCUR1 appear to be missing in both *Trypanosoma* and *Leishmania* spp [76].

Ca^2+^ release from mammalian mitochondria is mediated by the action of either a Na^+^/Ca^2+^ exchanger called NCLX [77] or a Ca^2+^/H^+^ exchanger. There is physiological evidence for the presence of a Ca^2+^/H^+^ exchanger in *T. cruzi* [1]. Trypanosomatids lack orthologs for NCLX but do contain orthologs for the leucine zipper EF hand-containing transmembrane protein 1 (Letm1) protein, a proposed Ca^2+^/H^+^ exchanger [78]. However, it has been proposed that the LETM1 ortholog functions as a K^+^/H^+^ exchanger in *T. brucei* [79]. Such a role for Letm1 in K^+^/H^+^ exchange has also been proposed in yeast and humans [80]. Recent work with LETM1 in mammalian cells suggests that Letm1 probably functions as a monovalent cation exchanger thereby functioning as a Na^+^/H^+^ or a K^+^/H^+^ exchanger. Changes in mitochondrial Ca^2+^ associated with Letm1 modulation may be due to its role in Na^+^/H^+^ exchange [81]. Whether this model holds true in trypanosomatids as well, remains to be determined.

## 6. Calcium Transport Proteins in the Membrane of Acidocalcisomes

Acidocalcisomes are electron dense acidic organelles that harbor a high concentration of phosphate, pyrophosphate, polyphosphate, magnesium and calcium [9]. Acidocalcisomes in trypanosomatids have received particular attention, as they are the largest calcium reservoir in these organisms [44]. The presence of Ca^2+^ in acidocalcisomes of *T. cruzi* was first detected by X-ray microanalysis [82]. The acidity of acidocalcisomes is maintained by the action of two proton pumps. A vacuolar type ATPase (V-ATPase) and a vacuolar pyrophosphatase (VP1) import H^+^ into these organelles by hydrolysis of ATP and pyrophosphate, respectively [83]. The H^+^ accumulated in the acidocalcisomes can then be used by either cation/H^+^ exchangers or a H^+^/Ca^2+^-ATPase [45,84,85] to import Ca^2+^ or other cations into the acidocalcisomes. For Ca^2+^ release, the trypanosomatid acidocalcisomes harbor an IP_3_R. In mammalian cells, this protein localizes to the ER. In *T. brucei* its unique localization in acidocalcisomes was initially observed by epitope tagging [10] and later confirmed by proteomic analysis and a IP_3_R specific antibody labeling [85] (Table 1). Furthermore, recent development of CRISPR/Cas9 based in situ epitope tagging in *T. cruzi* showed that the *T. cruzi* IP_3_R (TcIP_3_R) is also localized to the acidocalcisomes of the parasites [11]. In *T. brucei*, RNA interference (RNAi) mediated downregulation of IP_3_R reduces parasite growth in both procyclic and bloodstream forms. Additionally, it also reduces the ability of IP_3_ to release Ca^2+^ from permeabilized cells [10]. Deletion of the IP_3_R in *T. cruzi* was unsuccessful [86]. However, overexpression or reduced expression of TcIP_3_R results in reduced parasite proliferation, differentiation, and infectivity [86] indicating the significance of this protein in *T. cruzi*. This significance is further corroborated by the observation that reduced expression or activity of TcIP_3_R is associated with differentiation into trypomastigotes [87]. Moreover, functional studies with TbIP_3_R or TcIP_3_R in a chicken B lymphocyte cell line devoid of endogenous IP_3_Rs indicated that both these proteins could be stimulated by IP_3_ to release Ca^2+^ from permeabilized or intact cells [10,86]. Together these studies define a clear role for a functional IP_3_R in the acidocalcisomes of both *T. brucei* and *T. cruzi*.

## 7. An Emerging Role for Membrane Contact Sites in Ca^2+^ Signaling

The ER is the major Ca^2+^ storage site in mammalian cells [88]. As the ER ramifies throughout the entire cell body, it forms regions of close contact with various other organelles. Such regions of close contact wherein the organellar membranes are ≤ 30 nm apart are referred to as membrane contact sites (MCS). In mammalian cells such contact sites are often observed between ER-plasma membrane, ER-mitochondria and ER-endosomes, which facilitate direct transfer of Ca^2+^ and other biomolecules between these organelles without disturbing the overall cytosolic balance [89]. Similar membrane contact sites have also been observed in yeast [90] and plants [91]. However, their existence in trypanosomatids was not investigated until recently.

**Figure 1:**
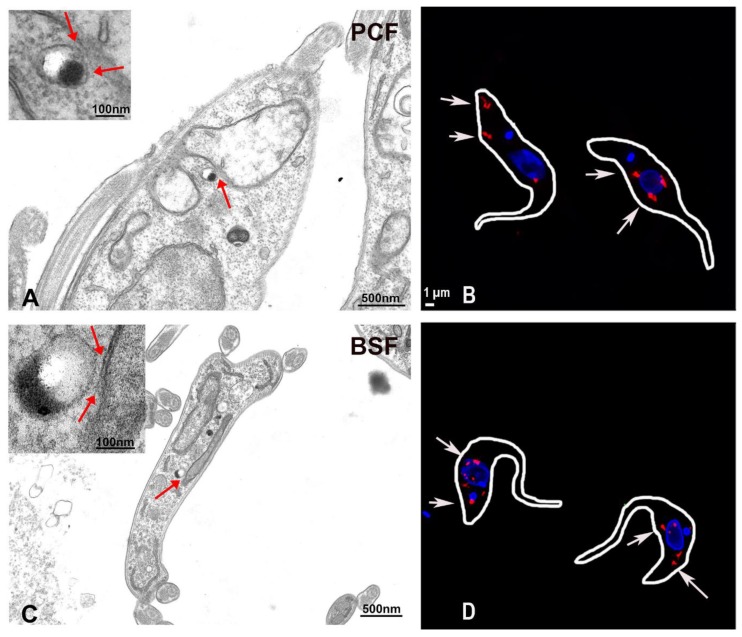
Representative transmission electron microscopy images of procyclic forms (PCF) (**A**) and bloodstream forms (BSF) (**C**) of *Trypanosoma brucei* showing contacts between acidocalcisomes and the mitochondria of the parasites. Acidocalcisomes appear as rounded organelles containing electron-dense material that adheres to one side of the membrane and are seen adjacent to the mitochondrion double membrane. The contact sites could be observed in both life cycle forms and are indicated by red arrows in the insets at higher magnification. Representative super resolution structured illumination images of *T. brucei* PCF (**B**) and BSF (**D**) trypanosomes subjected to proximity ligation assay. The red fluorescent signals indicated by the white arrows confirm the existence of membrane contact sites between acidocalcisomes and the mitochondria in these parasites. Only some of them are labeled with arrows. Scale bar in B also applies to D. Reproduced with permission from reference [92].

Among the various MCS, ER-mitochondria MCS are of particular interest. Ca^2+^ transfer to the mitochondria is necessary for cellular bioenergetics and for regulation of cell death processes [89]. Such significance of mitochondrial Ca^2+^ uptake has also been demonstrated in *T. brucei* wherein loss of the MCU affects the AMP/ATP ratio and stimulates autophagy [7]. Interestingly, acidocalcisomes, the main storage site for Ca^2+^ in trypanosomatids, also house the calcium release channel IP_3_R. Therefore, the role of mammalian ER-mitochondria MCS might be replaced by acidocalcisome-mitochondrion MCS in trypanosomes. In support of this hypothesis, our group recently demonstrated existence of such contact sites in *T. brucei*. Using high resolution microscopy and electron microscopy (Figure 1), close apposition between the parasite acidocalcisomes and mitochondrion could be observed. Additionally, such acidocalcisome-mitochondrion points of contact were clearly identified by using a proximity ligation assay [92].

Whether these contact sites participate in *T. brucei* mitochondrial Ca^2+^ uptake remains to be evaluated. However, this hypothesis is supported by a previous study. In this study, genetically encoded Ca^2+^ indicators targeted to the *T. brucei* mitochondrion suggested the existence of a selective route for transfer of Ca^2+^ from acidic stores to the parasite mitochondrion [93]. It is highly likely that acidocalcisome-mitochondrion MCS provide this selective route for Ca^2+^ transfer in the parasite. Also, whether these contact sites exist in *Leishmania* spp. and *T. cruzi* remains to be seen as well. However, the existence of these contact sites in *T. brucei* provides sufficient basis to investigate their existence in other trypanosomatids and to characterize their role in trypanosomatid Ca^2+^ signaling.

## 8. A Potential Role for the Golgi Complex in Ca^2+^ Signaling

In mammals Ca^2+^ uptake into the Golgi is mediated by SERCA as well as by a Golgi-specific ATPase termed the secretory pathway Ca^2+^-ATPase (SPCA) [94,95]. An ortholog for this protein in yeast is also identified and termed as Pmr1p [96]. A Golgi-specific ortholog for SPCA/Pmr1p proteins have not been found in either *Trypanosoma* or *Leishmania spp*. Additionally, trypanosomatids seem to lack orthologs for Golgi specific Ca^2+^ binding proteins such as Calnuc, Cab45 and Calumenin [97]. Therefore, it is not known if the Golgi complex can even function as a Ca^2+^ store in trypanosomatids. However, new proteins that play a role in Golgi ion homeostasis are still being discovered in mammals that could also be conserved in trypanosomatid parasites.

Recently a Golgi resident protein belonging to the unknown protein family 0016 (UPF0016 family), termed transmembrane protein 165 (TMEM165) in humans and Gcr1 dependent translation factor 1 (Gdt1p) in yeast, was suggested to be a Ca^2+^/H^+^ exchanger. Gdt1p was shown to be indispensable for growth of *Saccharomyces cerevisiae* under high Ca^2+^ concentrations. The human ortholog TMEM165 was able to rescue the growth phenotype associated with the loss of Gdt1p indicating functional conservation between these proteins [98,99,100]. Mutations in TMEM165 are associated with human congenital glycosylation disorders [101]. Further, Gdt1p is required for protein glycosylation in yeast indicating that this Golgi resident Ca^2+^ transporter could play an important role in protein glycosylation. However, more recent studies have indicated that although Gdt1p has higher affinity for Ca^2+^, it is also able to transport Mn^2+^ and that its role in Mn^2+^ homeostasis may be key for efficient protein glycosylation in the Golgi [101]. An ortholog for this protein seems to be present in *Leishmania* spp. and *T. cruzi* but cannot be found in the *T. brucei* genome (Table 1). These orthologs also retain the amino acid residues from the pore domain, which were shown to be indispensable for Ca^2+^ tolerance in yeast [98]. It is possible that these orthologs of Gdt1 function in Golgi Ca^2+^ homeostasis of trypanosomatids. However, their localization and role in these parasites awaits characterization.

Golgi specific Ca^2+^ release channels have not been identified yet. It is believed that ER localized IP_3_R [102] or RyR [103] also localize to Golgi and facilitate Ca^2+^ export. Since RyR are absent in trypanosomatids and the IP_3_R appears to localize to acidocalcisomes instead of ER and Golgi, it is currently unknown how the Ca^2+^ stored in trypanosomatid Golgi could be exported.

## 9. Calcium Transport Proteins in the Flagellar Membrane

The role of Ca^2+^ in the development of trypanosome flagellum was realized early when it was reported that chelation of Ca^2+^ resulted in detachment of the flagellum from the cell body of newly formed daughter cells [104]. Further research on a different trypanosomatid, *Crithidia oncopelti,* suggested that alterations in the extracellular calcium concentration can alter the flagellum wave pattern of this trypanosomatid [105]. Based on these results it was believed that the trypanosomatid flagellum can sense extracellular Ca^2+^ concentrations. In support of its role in Ca^2+^ signaling, trypanosomatid flagellum harbors several Ca^2+^ binding proteins. Centrins are well-known calcium binding proteins. Three centrins of *T. brucei* localize to the flagellar basal body [25,106]. Among these, an RNAi mediated knockdown of TbCen3 compromises cell motility [24]. *T. brucei* also contain a family of Ca^2+^ binding proteins that localize to the flagellum and are termed calflagins [107]. Their localization to the parasite flagellum and their ability to bind Ca^2+^ has been demonstrated experimentally. RNAi mediated knockdown of entire calflagin family does not reduce parasite motility but results in reduced parasitemia and increased mice survival rate during in vivo infection [108]. A related Ca^2+^-binding protein that localizes to the flagellum has been identified in *T. cruzi* [109]. This protein termed the flagellar calcium binding protein (FCaBP) employs a *N*-terminal myristoylation and palmitoylation signals to facilitate its transport to the flagellum [110]. Another family of Ca^2+^ binding proteins called calmodulins can also be found in trypanosomatids. At least one such calmodulin has been localized to the *T. brucei* flagellum [111] and is required for flagellar attachment and cell motility [112]. In addition to this calmodulin, there are four other flagellar proteins named paraflagellar rod components (PFC): TbPFC1, TbPFC6, TbPFC7 and Tb5.20, which have been suggested to have EF-hand domains indicating their potential to bind Ca^2+^ and regulate the development or activity of the flagellum [113]. The plasma membrane PMCA-ATPase also localizes to the flagellum of *T. brucei* [46]. Additionally, a putative Ca^2+^ channel, which localizes to the flagellum of *T. brucei*, has been demonstrated to be essential for parasite growth and flagellar attachment to the cell body [37]. Overall these evidences clearly outline the indispensable role for Ca^2+^ signaling in the development and maintenance of the trypanosomatid flagellum.

## 10. Ca^2+^ Binding Proteins

A major portion of Ca^2+^ binding proteins belong to the EF-hand superfamily. More than 800 different proteins have been assigned to this family. Proteins from this family contain at least one EF-hand domain, which is approximately 30 amino acids long, and forms the Ca^2+^ binding loop. Among the EF-domain containing proteins, CaM is the most conserved Ca^2+^ binding proteins and is found in all eukaryotes [114,115,116]. CaM from *T. cruzi* has been purified and shown to activate the plasma membrane Ca^2+^-ATPase [48] and cyclic AMP phosphodiesterase enzyme [114]. A similar study also characterized the role of *T. brucei* CaM in activating the plasma membrane Ca^2+^-ATPase [49]. The *T. brucei* CaM has been localized to the paraflagellar rod. RNAi mediated loss of this protein affected the paraflagellar rod assembly and caused the flagellum to detach from the cell body [112].

Interestingly, the *T. cruzi* CaM does not localize to the flagellum but to the spongiome of the contractile vacuole [117,118]. Several other CaM-like proteins with EF-hand domains have also been identified in the genome of trypanosomatids [40] but they remain to be characterized (Table 2). Other proteins with low capacity and high affinity for binding Ca^2+^ include the FCaBP in *T. cruzi* [109] and the calflagins in *T. brucei* [107]. The calflagins have been shown to be important for *T. brucei* infection in mice [108]. In addition to these, the genomes of trypanosomatids also contain several hypothetical proteins with Ca^2+^ binding domains, which remain to be characterized.

## 11. Conclusions and Future Directions

**Figure 2:**
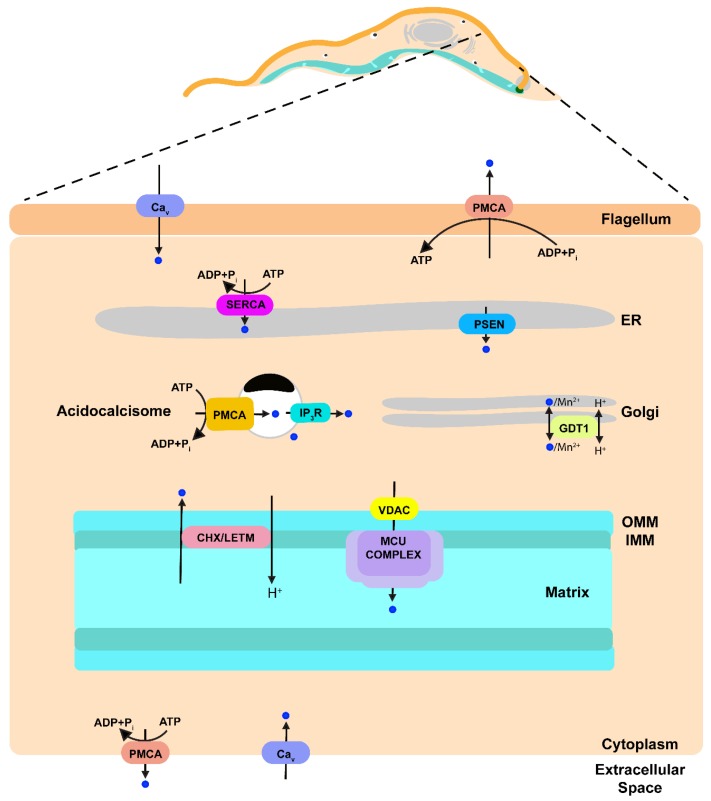
Schematic representation of Ca^2+^ transport pathways within a model trypanosomatid parasite based on data published previously. Ca^2+^ is indicated as blue circles. Ca^2+^ upon entering the cell through a Ca^2+^ channel can be sequestered into the ER by the action of SERCA, into the mitochondrion by the action of MCU complex or into the acidocalcisomes by the action of a PMCA. Ca^2+^ release from the mitochondrion is through a Ca^2+^/H^+^ exchanger (CHX) that could be LETM1. Ca^2+^ from acidocalcisomes is released when IP_3_ stimulates the IP_3_R located in this organelle. A presenilin can be a leak channel in the ER. Ca^2+^ release through the parasite plasma membrane is achieved by a PMCA. PMCA, plasma membrane Ca^2+^-ATPase; Ca_v_ Voltage gated Ca^2+^ channel; IP_3_, inositol 1,4,5-trisphosphate; IP_3_R, IP_3_ receptor; GDT1, GCR1 dependent translation factor; MCU complex, mitochondrial calcium uniporter complex; VDAC, voltage-dependent anion-selective channel; Letm1 (LETM), leucine zipper-EF-hand containing transmembrane protein 1; CHX, Ca^2+^/H^+^ exchanger; PSEN presenilin; SERCA, sarcoplasmic-endoplasmic reticulum Ca^2+^-ATPase; ER, endoplasmic reticulum; OMM, outer mitochondrial membrane; IMM, inner mitochondrial membrane.

Trypanosomatids have some differences and similarities with mammalian cells regarding Ca^2+^ homeostasis and signaling (Figure 2). Some of the similarities were important for the discovery of the molecular nature of the MCU complex in mammalian cells [3]. Some channels and pumps are present but with peculiar localizations: an IP_3_R is in the acidocalcisomes instead of in the endoplasmic reticulum, a voltage-dependent Ca^2+^ channel is present in the flagellum, and a PMCA localizes to the acidocalcisomes in addition to the plasma membrane. Some transporters are missing, such as plasma membrane and mitochondrial Na^+^/Ca^2+^ exchangers, several subunits (MICU3, MCR1, EMRE) of the mitochondrial Ca^2+^ uniporter, and the components of the SOCE mechanism.

The presence of TRP in acidic stores [42] and the importance of acidic stores in Ca^2+^ signaling [119] suggest that more studies of these channels are needed. The essentiality of different membrane transporters involved in Ca^2+^ homeostasis and signaling also suggest that they could be exploited as drug, vaccines, or diagnostic targets. In this regard, trypanosomatids, which belong to the Excavata supergroup of eukaryotes, diverged early from the Ophistonkonta, containing animals, and fungi, and the differences pointed out in Ca^2+^ homeostasis and signaling suggest that the search for new targets is warranted.

## Figures and Tables

**Table 1:** Organellar distribution of confirmed and putative Ca^2+^ signaling transporters in trypanosomatids.

Organelle	Protein	*Trypanosoma brucei*	*Trypanosoma cruzi*	*Leishmania major*
**Endoplasmic Reticulum (ER)**	SERCA	Tb927.5.3400	TcCLB.509777.70	LmjF.04.0010
		Tb927.9.15460	TcCLB.506241.70	LmjF.35.2080
	PSEN	Tb927.9.4940	TcCLB.508277.50	LmjF.15.1530
**Mitochondrion**	VDAC	Tb927.2.2510	TcCLB.504225.20	LmjF.02.0460
		Tb927.2.2520	TcCLB.508741.229	
			TcCLB.509141.40	
			TcCLB.511687.10	
			TcCLB.511687.10	
	VDAC like 2	Tb927.4.1610	TcCLB504057	LmjF.34.3100
	MCU	Tb927.11.1350	TcCLB.503893.120	LmjF.27.0780
	MICU1	Tb927.8.1850	TcCLB.511391.210	LmjF.07.0110
	MICU2	Tb927.7.2960	TcCLB.510525.130	
	MCUb	Tb927.10.300	TcCLB.504069	LmjF.21.1690
	LETM1	Tb927.3.4920	TcCLB.507951.270	LmjF.29.0920
**Acidocalcisome**	Ca^2+^ ATPase	Tb927.8.1180	TcCLB.508543.90	LmjF.07.0630
				LmjF.07.0650
	IP_3_R	Tb927.8.2770	TcCLB.509461.90	LmjF.16.0280
**Golgi**	Gdt1	-	TcCLB.508895.70	LmjF.19.0310
**Plasma membrane**	PMCA	Tb927.8.1180	TcCLB.506401.70	LmjF.07.0630
		Tb927.8.1160	TcCLB.508543.90	LmjF.07.0650
		Tb927.8.1200	TcCLB.510769.120	LmjF.17.0600
		Tb927.10.11620	TcCLB.509647.150	LmjF.33.1010
	Ca_v_ channel (flagellum)	Tb927.10.2880	TcCLB.504105.130	LmjF.34.0480
				LmjF.17.1440
**Acidic stores**	TRP	Tb927.8.850	TcCLB.510861.94	LmjF.07.0910.
	TRPML	Tb927.7.950	TcCLB.503463.20	LmjF.26.0990
			TcCLB.503735.30	

**Table 2:** Characterized and putative Ca^2+^ binding proteins in trypanosomatids.

Protein	*T. brucei*	*T. cruzi*	*L. major*
Calreticulin	Tb927.8.7410	TcCLB.509011.40	LmjF.31.2600
Flagellar Ca^2+^-binding protein	Tb927.8.5440	TcCLB.509391.10	LmjF.16.0910
	Tb927.8.5460	TcCLB.509391.20	LmjF.16.0920
	Tb927.8.5465	TcCLB.509391.30	
	Tb927.8.5470	TcCLB.506749.20	
Ca^2+^-binding protein	Tb927.6.2720	TcCLB.507925.60	LmjF.30.1240
	Tb927.4.1740	TcCLB.510879.190	LmjF.34.2950
Calmodulin (CaM)	Tb927.11.13020	TcCLB.507483.30	LmjF.09.0910
	Tb927.11.13030	TcCLB.507483.39	LmjF.09.0920
	Tb927.11.13040		LmjF.09.0930
	Tb927.11.13050		
CaM-like protein	Tb927.11.9790	TcCLB.506963.90	LmjF.36.3675
	Tb927.9.11230	TcCLB.504075.3	LmjF.35.3890
	Tb927.11.3680	TcCLB.508731.30	LmjF.13.1160
	Tb927.11.7940	TcCLB.506933.89	LmjF.28.0800
	Tb927.9.6130	TcCLB.508951.50	LmjF.21.0220
	Tb927.6.4710	TcCLB.511729.9	LmjF.15.0930
		TcCLB.507483.50	LmjF.30.3360

## References

[1] Docampo R., Vercesi A.E. (1989). Characteristics of Ca^2+^ transport by *Trypanosoma cruzi* mitochondria in situ. Arch. Biochem. Biophys..

[2] Docampo R., Vercesi A.E. (1989). Ca^2+^ transport by coupled *Trypanosoma cruzi* mitochondria in situ. J. Biol. Chem..

[3] Docampo R., Lukes J. (2012). Trypanosomes and the solution to a 50-year mitochondrial calcium mystery. Trends Parasitol..

[4] Perocchi F., Gohil V.M., Girgis H.S., Bao X.R., McCombs J.E., Palmer A.E., Mootha V.K. (2010). MICU1 encodes a mitochondrial EF hand protein required for Ca^2+^ uptake. Nature.

[5] Baughman J.M., Perocchi F., Girgis H.S., Plovanich M., Belcher-Timme C.A., Sancak Y., Bao X.R., Strittmatter L., Goldberger O., Bogorad R.L. (2011). Integrative genomics identifies MCU as an essential component of the mitochondrial calcium uniporter. Nature.

[6] De Stefani D., Raffaello A., Teardo E., Szabo I., Rizzuto R. (2011). A forty-kilodalton protein of the inner membrane is the mitochondrial calcium uniporter. Nature.

[7] Huang G., Vercesi A.E., Docampo R. (2013). Essential regulation of cell bioenergetics in *Trypanosoma brucei* by the mitochondrial calcium uniporter. Nat. Commun..

[8] Pan X., Liu J., Nguyen T., Liu C., Sun J., Teng Y., Fergusson M.M., Rovira I.I., Allen M., Springer D.A. (2013). The physiological role of mitochondrial calcium revealed by mice lacking the mitochondrial calcium uniporter. Nat. Cell Biol..

[9] Docampo R., de Souza W., Miranda K., Rohloff P., Moreno S.N. (2005). Acidocalcisomes—Conserved from bacteria to man. Nat. Rev. Microbiol..

[10] Huang G., Bartlett P.J., Thomas A.P., Moreno S.N., Docampo R. (2013). Acidocalcisomes of *Trypanosoma brucei* have an inositol 1,4,5-trisphosphate receptor that is required for growth and infectivity. Proc. Natl. Acad. Sci. USA.

[11] Lander N., Chiurillo M.A., Storey M., Vercesi A.E., Docampo R. (2016). CRISPR/Cas9-mediated endogenous C-terminal tagging of *Trypanosoma cruzi* genes reveals the acidocalcisome localization of the inositol 1,4,5-trisphosphate receptor. J. Biol. Chem..

[12] Carafoli E., Krebs J. (2016). Why calcium? How calcium became the best communicator. J. Biol. Chem..

[13] Collins K.D. (1997). Charge density-dependent strength of hydration and biological structure. Biophys. J..

[14] Parsons M., Ruben L. (2000). Pathways involved in environmental sensing in trypanosomatids. Parasitol. Today.

[15] Swulius M.T., Waxham M.N. (2008). Ca^2+^/calmodulin-dependent protein kinases. Cell. Mol. Life Sci..

[16] Nogueira N.P., de Souza C.F., Saraiva F.M., Sultano P.E., Dalmau S.R., Bruno R.E., Goncalves Rde L., Laranja G.A., Leal L.H., Coelho M.G. (2011). Heme-induced ros in *Trypanosoma cruzi* activates CaMKII-like that triggers epimastigote proliferation. One helpful effect of ROS. PLoS ONE.

[17] Souza C.F., Carneiro A.B., Silveira A.B., Laranja G.A., Silva-Neto M.A., Costa S.C., Paes M.C. (2009). Heme-induced *Trypanosoma cruzi* proliferation is mediated by CaM kinase II. Biochem. Biophys. Res. Commun..

[18] Ogueta S., Intosh G.M., Tellez-Iñon M.T. (1996). Regulation of Ca^2+^/calmodulin-dependent protein kinase from *Trypanosoma cruzi*. Mol. Biochem. Parasitol..

[19] Ogueta S.B., Macintosh G.C., Tellez-Iñon M.T. (1998). Stage-specific substrate phosphorylation by a Ca^2+^/calmodulin-dependent protein kinase in *Trypanosoma cruzi*. J. Eukaryot. Microbiol..

[20] Furuya T., Kashuba C., Docampo R., Moreno S.N. (2000). A novel phosphatidylinositol-phospholipase C of *Trypanosoma cruzi* that is lipid modified and activated during trypomastigote to amastigote differentiation. J. Biol. Chem..

[21] Lammel E.M., Barbieri M.A., Wilkowsky S.E., Bertini F., Isola E.L. (1996). *Trypanosoma cruzi*: Involvement of intracellular calcium in multiplication and differentiation. Exp. Parasitol..

[22] Stojdl D.F., Clarke M.W. (1996). *Trypanosoma brucei*: Analysis of cytoplasmic Ca^2+^ during differentiation of bloodstream stages in vitro. Exp. Parasitol..

[23] D’Angelo M.A., Montagna A.E., Sanguineti S., Torres H.N., Flawia M.M. (2002). A novel calcium-stimulated adenylyl cyclase from *Trypanosoma cruzi*, which interacts with the structural flagellar protein paraflagellar rod. J. Biol. Chem..

[24] Wei Y., Hu H., Lun Z.R., Li Z. (2014). Centrin3 in trypanosomes maintains the stability of a flagellar inner-arm dynein for cell motility. Nat. Commun..

[25] Selvapandiyan A., Kumar P., Morris J.C., Salisbury J.L., Wang C.C., Nakhasi H.L. (2007). Centrin1 is required for organelle segregation and cytokinesis in *Trypanosoma brucei*. Mol. Biol. Cell.

[26] Araya J.E., Cornejo A., Orrego P.R., Cordero E.M., Cortez M., Olivares H., Neira I., Sagua H., da Silveira J.F., Yoshida N. (2008). Calcineurin B of the human protozoan parasite *Trypanosoma cruzi* is involved in cell invasion. Microbes Infect..

[27] Selvapandiyan A., Debrabant A., Duncan R., Muller J., Salotra P., Sreenivas G., Salisbury J.L., Nakhasi H.L. (2004). Centrin gene disruption impairs stage-specific basal body duplication and cell cycle progression in *Leishmania*. J. Biol. Chem..

[28] Selvapandiyan A., Duncan R., Debrabant A., Bertholet S., Sreenivas G., Negi N.S., Salotra P., Nakhasi H.L. (2001). Expression of a mutant form of *Leishmania donovani* centrin reduces the growth of the parasite. J. Biol. Chem..

[29] Moreno V.R., Aguero F., Tekiel V., Sanchez D.O. (2007). The calcineurin a homologue from *Trypanosoma cruzi* lacks two important regulatory domains. Acta Trop..

[30] Moreno S.N., Silva J., Vercesi A.E., Docampo R. (1994). Cytosolic-free calcium elevation in *Trypanosoma cruzi* is required for cell invasion. J. Exp. Med..

[31] Lu H.G., Zhong L., Chang K.P., Docampo R. (1997). Intracellular Ca^2+^ pool content and signaling and expression of a calcium pump are linked to virulence in *Leishmania mexicana amazonesis* amastigotes. J. Biol. Chem..

[32] Yakubu M.A., Majumder S., Kierszenbaum F. (1994). Changes in *Trypanosoma cruzi* infectivity by treatments that affect calcium ion levels. Mol. Biochem. Parasitol..

[33] Rohloff P., Rodrigues C.O., Docampo R. (2003). Regulatory volume decrease in *Trypanosoma cruzi* involves amino acid efflux and changes in intracellular calcium. Mol. Biochem. Parasitol..

[34] Irigoin F., Inada N.M., Fernandes M.P., Piacenza L., Gadelha F.R., Vercesi A.E., Radi R. (2009). Mitochondrial calcium overload triggers complement-dependent superoxide-mediated programmed cell death in *Trypanosoma cruzi*. Biochem. J..

[35] Selzer P.M., Webster P., Duszenko M. (1991). Influence of Ca^2+^ depletion on cytoskeleton and nucleolus morphology in *Trypanosoma brucei*. Eur. J. Cell. Biol..

[36] Prole D.L., Taylor C.W. (2011). Identification of intracellular and plasma membrane calcium channel homologues in pathogenic parasites. PLoS ONE.

[37] Oberholzer M., Langousis G., Nguyen H.T., Saada E.A., Shimogawa M.M., Jonsson Z.O., Nguyen S.M., Wohlschlegel J.A., Hill K.L. (2011). Independent analysis of the flagellum surface and matrix proteomes provides insight into flagellum signaling in mammalian-infectious *Trypanosoma brucei*. Mol. Cell. Proteom..

[38] Benaim G., Garcia-Marchan Y., Reyes C., Uzcanga G., Figarella K. (2013). Identification of a sphingosine-sensitive Ca^2+^ channel in the plasma membrane of *Leishmania mexicana*. Biochem. Biophys. Res. Commun..

[39] Pinto-Martinez A.K., Rodriguez-Duran J., Serrano-Martin X., Hernandez-Rodriguez V., Benaim G. (2018). Mechanism of action of miltefosine on *Leishmania donovani* involves the impairment of acidocalcisome function and the activation of the sphingosine-dependent plasma membrane Ca^2+^ channel. Antimicrob. Agents Chemother..

[40] Docampo R., Huang G. (2015). Calcium signaling in trypanosomatid parasites. Cell Calcium.

[41] Taylor M.C., McLatchie A.P., Kelly J.M. (2013). Evidence that transport of iron from the lysosome to the cytosol in african trypanosomes is mediated by a mucolipin orthologue. Mol. Microbiol..

[42] Cruz-Bustos T., Moreno S.N.J., Docampo R. (2018). Detection of weakly expressed *Trypanosoma cruzi* membrane proteins using high-performance probes. J. Eukaryot. Microbiol..

[43] Prakriya M., Lewis R.S. (2015). Store-operated calcium channels. Physiol. Rev..

[44] Ulrich P., Cintron R., Docampo R., Souza W.D. (2010). Calcium homeostasis and acidocalcisomes in *Trypanosoma cruzi*. Structures and Organelles in Pathogenic Protists.

[45] Lu H.G., Zhong L., de Souza W., Benchimol M., Moreno S., Docampo R. (1998). Ca^2+^ content and expression of an acidocalcisomal calcium pump are elevated in intracellular forms of *Trypanosoma cruzi*. Mol. Cell. Biol..

[46] Luo S., Rohloff P., Cox J., Uyemura S.A., Docampo R. (2004). *Trypanosoma brucei* plasma membrane-type Ca^2+^-ATPase 1 (TbPMC1) and 2 (TbPMC2) genes encode functional Ca^2+^-ATPases localized to the acidocalcisomes and plasma membrane, and essential for Ca^2+^ homeostasis and growth. J. Biol. Chem..

[47] Mandal D., Mukherjee T., Sarkar S., Majumdar S., Bhaduri A. (1997). The plasma-membrane Ca^2+^-ATPase of *Leishmania donovani* is an extrusion pump for Ca^2+^. Biochem. J..

[48] Benaim G., Losada S., Gadelha F.R., Docampo R. (1991). A calmodulin-activated (Ca^2+^-Mg^2+^)-ATPase is involved in Ca^2+^ transport by plasma membrane vesicles from *Trypanosoma cruzi*. Biochem. J..

[49] Benaim G., Cervino V., Hermoso T., Felibert P., Laurentin A. (1993). Intracellular calcium homeostasis in *Leishmania mexicana*. Identification and characterization of a plasma membrane calmodulin-dependent Ca^2+^-ATPase. Biol Res.

[50] Ramirez-Iglesias J.R., Perez-Gordones M.C., Del Castillo J.R., Mijares A., Benaim G., Mendoza M. (2018). Identification and characterization of a calmodulin binding domain in the plasma membrane Ca^2+^-ATPase from *Trypanosoma equiperdum*. Mol. Biochem. Parasitol..

[51] Perez-Gordones M.C., Ramirez-Iglesias J.R., Cervino V., Uzcanga G.L., Benaim G., Mendoza M. (2017). Evidence of the presence of a calmodulin-sensitive plasma membrane Ca^2+^-ATPase in *Trypanosoma equiperdum*. Mol. Biochem. Parasitol..

[52] Lai D.H., Hashimi H., Lun Z.R., Ayala F.J., Lukes J. (2008). Adaptations of *Trypanosoma brucei* to gradual loss of kinetoplast DNA: *Trypanosoma equiperdum* and *Trypanosoma evansi* are petite mutants of *T. brucei*. Proc. Natl. Acad. Sci. USA.

[53] Nolan D.P., Reverlard P., Pays E. (1994). Overexpression and characterization of a gene for a Ca^2+^-ATPase of the endoplasmic reticulum in *Trypanosoma brucei*. J. Biol. Chem..

[54] Mendoza M., Mijares A., Rojas H., Colina C., Cervino V., DiPolo R., Benaim G. (2004). Evaluation of the presence of a thapsigargin-sensitive calcium store in trypanosomatids using *Trypanosoma evansi* as a model. J. Parasitol..

[55] Furuya T., Okura M., Ruiz F.A., Scott D.A., Docampo R. (2001). TcSCA complements yeast mutants defective in Ca^2+^ pumps and encodes a Ca^2+^-ATPase that localizes to the endoplasmic reticulum of *Trypanosoma cruzi*. J. Biol. Chem..

[56] Conte I., Labriola C., Cazzulo J.J., Docampo R., Parodi A.J. (2003). The interplay between folding-facilitating mechanisms in *Trypanosoma cruzi* endoplasmic reticulum. Mol. Biol. Cell.

[57] Ramirez G., Valck C., Aguilar L., Kemmerling U., Lopez-Munoz R., Cabrera G., Morello A., Ferreira J., Maya J.D., Galanti N. (2012). Roles of *Trypanosoma cruzi* calreticulin in parasite-host interactions and in tumor growth. Mol. Immunol..

[58] Rimoldi M.T., Tenner A.J., Bobak D.A., Joiner K.A. (1989). Complement component C1q enhances invasion of human mononuclear phagocytes and fibroblasts by *Trypanosoma cruzi* trypomastigotes. J. Clin. Investig..

[59] Ramirez-Toloza G., Abello P., Ferreira A. (2016). Is the antitumor property of *Trypanosoma cruzi* infection mediated by its calreticulin?. Front. Immunol..

[60] Joshi M., Pogue G.P., Duncan R.C., Lee N.S., Singh N.K., Atreya C.D., Dwyer D.M., Nakhasi H.L. (1996). Isolation and characterization of *Leishmania donovani* calreticulin gene and its conservation of the RNA binding activity. Mol. Biochem. Parasitol..

[61] Debrabant A., Lee N., Dwyer D.M., Nakhasi H.L., Eggleton P.M.M. (2003). Role of calreticulin in *Leishmania* parasite secretory pathway and pathogenesis. Calreticulin.

[62] Honarnejad K., Herms J. (2012). Presenilins: Role in calcium homeostasis. Int. J. Biochem. Cell Biol..

[63] Denton R.M. (2009). Regulation of mitochondrial dehydrogenases by calcium ions. Biochim. Biophys. Acta.

[64] Denton R.M., Randle P.J., Martin B.R. (1972). Stimulation by calcium ions of pyruvate dehydrogenase phosphate phosphatase. Biochem. J..

[65] McCormack J.G., Denton R.M. (1979). The effects of calcium ions and adenine nucleotides on the activity of pig heart 2-oxoglutarate dehydrogenase complex. Biochem. J..

[66] Gincel D., Zaid H., Shoshan-Barmatz V. (2001). Calcium binding and translocation by the voltage-dependent anion channel: A possible regulatory mechanism in mitochondrial function. Biochem. J..

[67] Raffaello A., De Stefani D., Sabbadin D., Teardo E., Merli G., Picard A., Checchetto V., Moro S., Szabo I., Rizzuto R. (2013). The mitochondrial calcium uniporter is a multimer that can include a dominant-negative pore-forming subunit. EMBO J..

[68] Plovanich M., Bogorad R.L., Sancak Y., Kamer K.J., Strittmatter L., Li A.A., Girgis H.S., Kuchimanchi S., De Groot J., Speciner L. (2013). MICU2, a paralog of MICU1, resides within the mitochondrial uniporter complex to regulate calcium handling. PLoS ONE.

[69] Sancak Y., Markhard A.L., Kitami T., Kovacs-Bogdan E., Kamer K.J., Udeshi N.D., Carr S.A., Chaudhuri D., Clapham D.E., Li A.A. (2013). EMRE is an essential component of the mitochondrial calcium uniporter complex. Science.

[70] Mallilankaraman K., Cardenas C., Doonan P.J., Chandramoorthy H.C., Irrinki K.M., Golenar T., Csordas G., Madireddi P., Yang J., Muller M. (2012). MCUR1 is an essential component of mitochondrial Ca^2+^ uptake that regulates cellular metabolism. Nat. Cell Biol..

[71] Paupe V., Prudent J., Dassa E.P., Rendon O.Z., Shoubridge E.A. (2015). Ccdc90a (MCUR1) is a cytochrome c oxidase assembly factor and not a regulator of the mitochondrial calcium uniporter. Cell Metab..

[72] Vais H., Tanis J.E., Muller M., Payne R., Mallilankaraman K., Foskett J.K. (2015). MCUR1, CCDC90A, is a regulator of the mitochondrial calcium uniporter. Cell Metab..

[73] Pusnik M., Charriere F., Maser P., Waller R.F., Dagley M.J., Lithgow T., Schneider A. (2009). The single mitochondrial porin of *Trypanosoma brucei* is the main metabolite transporter in the outer mitochondrial membrane. Mol. Biol. Evol..

[74] Flinner N., Schleiff E., Mirus O. (2012). Identification of two voltage-dependent anion channel-like protein sequences conserved in kinetoplastida. Biol. Lett..

[75] Chiurillo M.A., Lander N., Bertolini M.S., Storey M., Vercesi A.E., Docampo R. (2017). Different roles of mitochondrial calcium uniporter complex subunits in growth and infectivity of *Trypanosoma cruzi*. MBio.

[76] Docampo R., Vercesi A.E., Huang G. (2014). Mitochondrial calcium transport in trypanosomes. Mol. Biochem. Parasitol..

[77] Palty R., Silverman W.F., Hershfinkel M., Caporale T., Sensi S.L., Parnis J., Nolte C., Fishman D., Shoshan-Barmatz V., Herrmann S. (2010). NCLX is an essential component of mitochondrial Na^+^/Ca^2+^ exchange. Proc. Natl. Acad. Sci. USA.

[78] Jiang D., Zhao L., Clapham D.E. (2009). Genome-wide RNAi screen identifies LETM1 as a mitochondrial Ca^2+^/H^+^ antiporter. Science.

[79] Hashimi H., McDonald L., Stribrna E., Lukes J. (2013). Trypanosome Letm1 protein is essential for mitochondrial potassium homeostasis. J. Biol. Chem..

[80] Froschauer E., Nowikovsky K., Schweyen R.J. (2005). Electroneutral K^+^/H^+^ exchange in mitochondrial membrane vesicles involves YOL027/Letm1 proteins. Biochim. Biophys. Acta.

[81] Austin S., Tavakoli M., Pfeiffer C., Seifert J., Mattarei A., De Stefani D., Zoratti M., Nowikovsky K. (2017). Letm1-mediated K^+^ and Na^+^ homeostasis regulates mitochondrial Ca^2+^ efflux. Front. Physiol..

[82] Dvorak J.A., Engel J.C., Leapman R.D., Swyt C.R., Pella P.A. (1988). *Trypanosoma cruzi*: Elemental composition heterogeneity of cloned stocks. Mol. Biochem. Parasitol..

[83] Scott D.A., Docampo R. (1998). Two types of H^+^-ATPase are involved in the acidification of internal compartments in *Trypanosoma cruzi*. Biochem. J..

[84] Docampo R., Scott D.A., Vercesi A.E., Moreno S.N. (1995). Intracellular Ca^2+^ storage in acidocalcisomes of *Trypanosoma cruzi*. Biochem. J..

[85] Huang G., Docampo R. (2015). Proteomic analysis of acidocalcisomes of *Trypanosoma brucei* uncovers their role in phosphate metabolism, cation homeostasis, and calcium signaling. Commun. Integr. Biol..

[86] Hashimoto M., Enomoto M., Morales J., Kurebayashi N., Sakurai T., Hashimoto T., Nara T., Mikoshiba K. (2013). Inositol 1,4,5-trisphosphate receptor regulates replication, differentiation, infectivity and virulence of the parasitic protist *Trypanosoma cruzi*. Mol. Microbiol..

[87] Hashimoto M., Morales J., Uemura H., Mikoshiba K., Nara T. (2015). A novel method for inducing amastigote-to-trypomastigote transformation in vitro in *Trypanosoma cruzi* reveals the importance of inositol 1,4,5-trisphosphate receptor. PLoS ONE.

[88] Koch G.L. (1990). The endoplasmic reticulum and calcium storage. Bioessays.

[89] Phillips M.J., Voeltz G.K. (2016). Structure and function of ER membrane contact sites with other organelles. Nat. Rev. Mol. Cell. Biol..

[90] Lang A., John Peter A.T., Kornmann B. (2015). ER-mitochondria contact sites in yeast: Beyond the myths of ERMES. Curr. Opin. Cell. Biol..

[91] Wang P., Hawes C., Hussey P.J. (2017). Plant endoplasmic reticulum-plasma membrane contact sites. Trends Plant Sci..

[92] Ramakrishnan S., Asady B., Docampo R. (2018). Acidocalcisome-mitochondrion membrane contact sites in *Trypanosoma brucei*. Pathogens.

[93] Xiong Z.H., Ridgley E.L., Enis D., Olness F., Ruben L. (1997). Selective transfer of calcium from an acidic compartment to the mitochondrion of *Trypanosoma brucei.* Measurements with targeted aequorins. J. Biol. Chem..

[94] Xiang M., Mohamalawari D., Rao R. (2005). A novel isoform of the secretory pathway Ca^2+^,Mn^2+^-ATPase, HsPCA2, has unusual properties and is expressed in the brain. J. Biol. Chem..

[95] Vanoevelen J., Dode L., Van Baelen K., Fairclough R.J., Missiaen L., Raeymaekers L., Wuytack F. (2005). The secretory pathway Ca^2+^/Mn^2+^-ATPase 2 is a Golgi-localized pump with high affinity for Ca^2+^ ions. J. Biol. Chem..

[96] Sorin A., Rosas G., Rao R. (1997). PMR1, a Ca^2+^-ATPase in yeast Golgi, has properties distinct from sarco/endoplasmic reticulum and plasma membrane calcium pumps. J. Biol. Chem..

[97] Pizzo P., Lissandron V., Capitanio P., Pozzan T. (2011). Ca^2+^ signalling in the Golgi apparatus. Cell Calcium.

[98] Colinet A.S., Thines L., Deschamps A., Flemal G., Demaegd D., Morsomme P. (2017). Acidic and uncharged polar residues in the consensus motifs of the yeast Ca^2+^ transporter GDT1p are required for calcium transport. Cell. Microbiol..

[99] Colinet A.S., Sengottaiyan P., Deschamps A., Colsoul M.L., Thines L., Demaegd D., Duchene M.C., Foulquier F., Hols P., Morsomme P. (2016). Yeast GDT1 is a Golgi-localized calcium transporter required for stress-induced calcium signaling and protein glycosylation. Sci. Rep..

[100] Demaegd D., Foulquier F., Colinet A.S., Gremillon L., Legrand D., Mariot P., Peiter E., Van Schaftingen E., Matthijs G., Morsomme P. (2013). Newly characterized Golgi-localized family of proteins is involved in calcium and pH homeostasis in yeast and human cells. Proc. Natl. Acad. Sci. USA.

[101] Dulary E., Potelle S., Legrand D., Foulquier F. (2017). TMEM165 deficiencies in congenital disorders of glycosylation type II (CDG-II): Clues and evidences for roles of the protein in Golgi functions and ion homeostasis. Tissue Cell.

[102] Micaroni M. (2012). Calcium around the Golgi apparatus: Implications for intracellular membrane trafficking. Adv. Exp. Med. Biol..

[103] Cifuentes F., Gonzalez C.E., Fiordelisio T., Guerrero G., Lai F.A., Hernandez-Cruz A. (2001). A ryanodine fluorescent derivative reveals the presence of high-affinity ryanodine binding sites in the golgi complex of rat sympathetic neurons, with possible functional roles in intracellular Ca^2+^ signaling. Cell. Signal..

[104] Vickerman K. (1969). On the surface coat and flagellar adhesion in trypanosomes. J. Cell Sci..

[105] Sugrue P., Hirons M.R., Adam J.U., Holwill M.E. (1988). Flagellar wave reversal in the kinetoplastid flagellate *Crithidia oncopelti*. Biol. Cell.

[106] Selvapandiyan A., Kumar P., Salisbury J.L., Wang C.C., Nakhasi H.L. (2012). Role of centrins 2 and 3 in organelle segregation and cytokinesis in *Trypanosoma brucei*. PLoS ONE.

[107] Wu Y., Deford J., Benjamin R., Lee M.G., Ruben L. (1994). The gene family of EF-hand calcium-binding proteins from the flagellum of *Trypanosoma brucei*. Biochem. J..

[108] Emmer B.T., Daniels M.D., Taylor J.M., Epting C.L., Engman D.M. (2010). Calflagin inhibition prolongs host survival and suppresses parasitemia in *Trypanosoma brucei* infection. Eukaryot. Cell.

[109] Engman D.M., Krause K.H., Blumin J.H., Kim K.S., Kirchhoff L.V., Donelson J.E. (1989). A novel flagellar Ca^2+^-binding protein in trypanosomes. J. Biol. Chem..

[110] Wingard J.N., Ladner J., Vanarotti M., Fisher A.J., Robinson H., Buchanan K.T., Engman D.M., Ames J.B. (2008). Structural insights into membrane targeting by the flagellar calcium-binding protein (FCaBP), a myristoylated and palmitoylated calcium sensor in *Trypanosoma cruzi*. J. Biol. Chem..

[111] Ridgley E., Webster P., Patton C., Ruben L. (2000). Calmodulin-binding properties of the paraflagellar rod complex from *Trypanosoma brucei*. Mol. Biochem. Parasitol..

[112] Ginger M.L., Collingridge P.W., Brown R.W., Sproat R., Shaw M.K., Gull K. (2013). Calmodulin is required for paraflagellar rod assembly and flagellum-cell body attachment in trypanosomes. Protist.

[113] Portman N., Lacomble S., Thomas B., McKean P.G., Gull K. (2009). Combining RNA interference mutants and comparative proteomics to identify protein components and dependences in a eukaryotic flagellum. J. Biol. Chem..

[114] Tellez-Inon M.T., Ulloa R.M., Torruella M., Torres H.N. (1985). Calmodulin and Ca^2+^-dependent cyclic AMP phosphodiesterase activity in *Trypanosoma cruzi*. Mol. Biochem. Parasitol..

[115] Wayne A Snedden H.F. (2001). Calmodulin as a versatile calcium signal transducer in plants. New Phytol..

[116] Villalobo A., Ishida H., Vogel H.J., Berchtold M.W. (2018). Calmodulin as a protein linker and a regulator of adaptor/scaffold proteins. Biochim. Biophys. Acta.

[117] Rohloff P., Montalvetti A., Docampo R. (2004). Acidocalcisomes and the contractile vacuole complex are involved in osmoregulation in *Trypanosoma cruzi*. J. Biol. Chem..

[118] Ulrich P.N., Jimenez V., Park M., Martins V.P., Atwood J., Moles K., Collins D., Rohloff P., Tarleton R., Moreno S.N. (2011). Identification of contractile vacuole proteins in *Trypanosoma cruzi*. PLoS ONE.

[119] Patel S., Docampo R. (2010). Acidic calcium stores open for business: Expanding the potential for intracellular Ca^2+^ signaling. Trends Cell Biol..

